# Research on the Resilience Evaluation and Spatial Correlation of China’s Sports Regional Development Under the New Concept

**DOI:** 10.3389/fpsyg.2021.763501

**Published:** 2022-02-04

**Authors:** Jing Zhang, Jing-Ru Gan, Ying Wu, Jia-Bao Liu, Su Zhang, Bin Shao

**Affiliations:** ^1^School of Physical Education and Training, Shanghai University of Sport, Shanghai, China; ^2^Faculty of Physical Education, Shanghai International Studies University, Shanghai, China; ^3^School of Statistics and Applied Mathematics, Anhui University of Finance and Economics, Bengbu, China; ^4^School of Mathematics and Physics, Anhui Jianzhu University, Hefei, China; ^5^Department of Physical Education, Anhui University of Finance and Economics, Bengbu, China

**Keywords:** new concept, resilience of sports development, DPSIR model, TOPSIS method, obstacle degree, spatial correlation

## Abstract

In order to fully implement the new development concept, bring into full play the potential of sports development, and maintain the resilience of China’s sports development. This paper studies the resilience evaluation and spatial correlation of Chinese sports development under the new development concept. First, we constructed Resilience Evaluation Indexes System for Sports Development in China based on the analysis of the resilience features of sports development and the DPSIR model, which is from the five aspects of “driving force – pressure – state – influence – response.” Second, used Coefficient of Variation and Technique for Order Preference by Similarity to an Ideal Solution (TOPSIS) Method to measure the resilience level of sports development in 31 provinces in China from 2013 to 2017. Then, we introduced the obstacle degree model to identify the obstacle factors that hinder the resilience of Chinese sports development in different periods. Finally, we used the global and local Moran indexes to analyze the spatial correlation of China sports regional development. The results showed that: (1) overall, the development level of sports resilience in 31 provinces in China showed an upward trend from 2013 to 2017, while some provinces showed obvious fluctuations. (2) The obstacles to the development of sports resilience in China mainly include sports scientific research equipment, the number of national fitness monitoring stations, the number of national fitness centers, the full-time equivalent of (R&D) personnel, and the number of sports scientific research projects. The response subsystem is the main obstacle factor that affects the improvement of the resilience level of sports development in China. (3) There is a positive spatial autocorrelation between the resilience level of sports development and regional spatial distribution, and the correlation shows a weakening trend, and the internal difference is significant. Finally, we concluded that we must take the new development philosophy as the guiding principle. First, we should stick to innovation-driven development to fully upgrade the resilience of China’s sports development. Second, we should adhere to the principle of coordinated development to promote the overall and balanced development of sports. Lastly, we should promote shared development so as to deliver benefits for all in an equal way.

## Introduction

With the development of economic globalization, sports have become an important criterion for measuring the development level of a country or a region. China has made remarkable achievements in the transformation, in which China became a sports great power not only in quantity but also in quality (Liu, 2021).

In recent years, the emergence of new situations such as the global financial crisis, political crisis, and climate change, especially the COVID-19 pandemic, has accelerated the occurrence of risks, and the world is facing greater risks and uncertainties (Adger, 2006). Some major risks and emergencies seriously threaten people’s physical and mental health and may have a major impact on global economic development and social stability. Therefore, resilience theory has been widely applied in multidisciplinary fields such as social ecology, economics, management, and urban planning, and abundant research results have been achieved in social resilience, regional resilience, and urban resilience (Xu et al., 2017).

At the time of the “14th Five-Year Plan,” the new phase of China’s development was not hindered by the epidemic, instead, it burst out with stronger development momentum, demonstrating resilience and strength of development, showing China’s strong restorability and vitality. This “resilience” is reflected in a variety of fields, including sports.

As China enters a new stage of development, with the approach of the Winter Olympics, new development concepts must be carried out completely, accurately, and comprehensively throughout the development and in the field of sports. While emphasizing sports development planning, we should also pay more attention to the transformation of sports development, gather reform momentum, promote practical development, promote the full potential of sports development, continue to maintain the resilience of China’s sports development, and take greater action in the future. Therefore, the study of human social development from the perspective of sports adaptation is a promising research field to find more effective coping strategies.

## Review of the Theory About Resilience or Related Research

The concept of resilience firstly appeared in the field of physics and was raised by Alexander, referring to the capability of a strained body to restore its size and shape after deformation caused especially by compressive stress (Holling, 1973). In 1973, Canadian ecologist Holling firstly introduced the concept of resilience to the field of ecology and applied it to the research of systemic questions. The concept of resilience has evolved from “engineering resilience” to “ecosystem resilience,” then to “socio-ecological system resilience” (Ni and Li, 2019). “Engineering resilience” emphasizes equilibrium of system and equilibrium state and believes that the system has only one equilibrium state; “ecological resilience” goes beyond the limit of single equilibrium of engineering resilience, based on ecological theory, emphasizes the connection between resilience and system, and believes that resilience can further promote new equilibrium based on restoring the equilibrium of the system. The concept of “socio-ecological resilience” extends the concept of resilience from natural ecosystem to social ecosystem, emphasizing not only the restoration of resilience balance and new equilibrium state but also the ability of the system to resist perturbations in the complex social ecosystem and to adapt to new circumstances. In other words, the system can repair, adapt, and change itself (Gunderson et al., 1995; Walker et al., 2004; Folke, 2006). In the evolution from engineering resilience, ecological resilience to socio-ecological resilience, the concept of resilience, system characteristics, and essential goals have changed. The connotation and extension of the concept of resilience have become more enriched, and the study of resilience theory has also become more fleshed out. Research on resilience, vulnerability, adaptability, and resilience has become the focus of attention in the fields of global change, disaster prevention and mitigation, and sustainable development. In particular, the topic of “resilience” has received increasing attention from society (Leichenko, 2011). Sport is a huge and complex system with external “driver” and internal “dynamism.” Once a sport system is impacted by external shocks, external drive decreases, or internal adaptability decreases, the sport system may stagnate or collapse (Zhu, 2014).

The research on resilience extends from psychology (psychological resilience) to management (organizational resilience), sociology (social resilience), and planning (urban and regional resilience). With the development of resilience theory, the concept of resilience has become richer in connotation and extension, and the research of resilience theory has also become more abundant. The research of resilience theory in sports systems is a new topic in recent years. Fletcher and Wagstaff (2009) and Paul et al. (2013) believe that sports organizations are characterized by highly complex social and organizational environments, and organizational resilience is likely to have a positive impact not only on the operation of sports organizations themselves but also on the resilience of individuals and teams. Kirsten et al. (2021) proposed a definition of organizational resilience applicable to elite sports organizations and determined the characteristics of elite sports organizations’ resilience, which provided an important basis for future research and practice in this field. Wang and Sun (2020) believe that the construction of stadium facilities should be adjusted in time with different stages of urban development to enhance the resilience of stadium facilities and improve their long-term adaptability (Hu et al., 2020). Ling et al. (2020) believe that enhanced stadium operation resilience can deal with the sudden risk crisis of large stadiums, effectively resist and absorb disturbance, and achieve adaptive development (Ling et al., 2020).

## The Influence of Sports on Social Development

The development includes individual development, organization development, and social development. Sustainable development should be achieved for individuals, organizations, entire regions, or societies. The sustainable development of the three is both systematic and interactive. Individuals are nested in organizations, which in turn are nested in a certain region or society. There must be mutual influence and interaction among regions, organizations, and individuals.

With the increase of capital strength and the need for cultural communication in the process of urbanization, sports have become one of the important ways to promote regional modernization, social development, and economic growth. On the one hand, it can set up a national or regional image and improve government capacity (Yu, 2002). On the other hand, it can optimize the economic structure and promote the improvement of regional infrastructure (Gratton and Henry, 2001; Kaplanidou and Karadakis, 2010). At the same time, the development of sports can improve population literacy, for example, a successful sports event can also make people generate good “psychological income,” such as increasing citizens’ sense of pride and honor (Cao and Lei, 2010; Shi et al., 2014).

Although individuals, organizations, and regions are separate systems, they are all complex and organized wholes. When facing the adversity caused by unexpected events, the region, organization, and individual need emergency linkage to minimize the loss caused by unexpected events. The development of sports can effectively enhance regional or social capital, which is conducive to the linkage and cooperation between the government, enterprises, social organizations, and residents. Therefore, a social network of common governance can be formed to effectively cope with social crises and improve the resilience of urban society, which is consistent with the resilience construction of urban society (Yan and Li, 2021). This study will explore effective coping strategies for the development of human society from the perspective of regional sports resilience by combining qualitative and quantitative methods.

## Construction of a Resilience Evaluation Indexes System for Sports Regional Development in China Based on the Driving Force – Pressure – State – Influence – Response Model

### The Driving Force – Pressure – State – Influence – Response Model

The “driving force – pressure – state – influence – response” (DPSIR) framework model was developed by the European Environment Agency in 1993 based on the PSR and DSR models. This model has inherited the advantages of PSR and DSR in constructing index systems using the “cause-effect-response” method and includes four major elements: economy, society, environment, and policy, which can greatly show the relationship between the external environment and human activities (Niemeijer and Groot, 2008; Svarstad et al., 2008).

Although DPSIR conceptual model is an important theory widely used in environmental and ecological governance, it is also suitable for the study of sports resilience development. Firstly, sport is a complex giant system with an open structure in nature, which is affected by both external environment and internal factors (Shao and Man, 2010; Chen, 2013; Shao, 2015). Secondly, the DPSIR model can show the interaction between humans and the environment from the perspective of the system life cycle, reflect the stage characteristics of resilience, and better reflect the timeliness and compatibility of resilience applied to the current regional sports development research. Finally, although those specific indexes of this model characterize human beings’ different feedbacks to environmental systems in dynamic activities, its cyclical approach fits with the evolutionary mechanism of resilience when the sport is disturbed in the development.

Therefore, it is reasonable to apply the DPSIR model to the evaluation of regional sports resilience development in this research, to show the relationship between human and environment interaction from the perspective of the system life cycle, and to construct an evaluation index system based on the process sequence of regional sports resilience stage.

In the DPSIR model, the driver (D) refers to the economic, social, and natural drivers of change and development in sports resilience, and is the initial index of change in sports resilience; the pressure (P) refers to indexes, under the effect of drivers, that directly exert pressure within the sport system so that changes are caused in urban competitive sports resilience; the state (S) refers to the real performance of sports resilience under the effect of drivers and pressures; the impact (I) refers to the economic, social, and natural effects of change in sports resilience; and the response (R) refers to the action taken in the face of change in sports resilience. State (S) refers to the actual performance of sports resilience under the driver and pressure; Impact (I) refers to the effect on economic, social, and natural aspects when sports resilience changes; Response (R) refers to the effective measures and countermeasures to be taken when sports resilience changes. Based on the DPSIR model, following the principles of comparability, operability, and incompatibility, considering the availability of data, referring to the research results of related literature (Li et al., 2020; Wang and Man, 2020), and at the same time, considering the actual situation of sports resilience development, 27 indexes were selected from five aspects: driver, pressure, state, impact, and response, to build an evaluation index system, as shown in Table 1.

**TABLE 1:** Resilience evaluation indexes system for sports regional development in China.

Target layer	Guideline layer	Index layer	unit	Indicator symbols
Driver	External drivers	GDP(+)	Billion yuan	D_1_
		Year-end population(+)	10,000	D_2_
		per capita disposable income(+)	Yuan	D_3_
		(R&D) personnel full-time equivalent(+)	FTE	D_4_
	Internal drivers	Culture, Sports and Media Financial Expenditure(+)	Billion	D_5_
		sports industry practitioners(+)	people	D_6_
		Numbers of public sports facilities(+)	PCS	D_7_
Pressure(P)	Economical pressure	GDP growth rate(+)	%	P_1_
	Population pressure	Natural population growth rate(+)	%	P_2_
	Social pressure	Unemployment rate	%	P_3_
	Natural resource pressure	Green coverage of built-up(+)	%	P_4_
State(S)	Competitive sports	Numbers of athletes of excellent sports teams(+)	people	S_1_
	Mass sports	rate of national reaching standard for physical quality measuring(+)	%	S_2_
		number of sports social organizations(+)	PCS	S_3_
	Sports industry	Sports lottery sales(+)	10,000yuan	S_4_
Impact(I)	Economic impact	value added of tertiary industry(+)	Hundred million yuan	I_1_
	Social impact	Employment in culture, sports and entertainment (+)	10,000 people	I_2_
	Demographic impact	Death rate	%	I_3_
	Natural resource impact	Forrest coverage(+)	%	I_4_
Response(R)	Early warning ability	Number of mobile internet users(+)	10,000 people	R_1_
	Restorability	Number of graded athletes in development(+)	people	R_2_
		Number of reserve sports talents(+)	people	R_3_
		Number of youth sports clubs(+)	PCS	R_4_
		number of national physical quality monitoring stations(+)	PCS	R_5_
		number of public fit-trail projects(+)	PCS	R_6_
	Learning and innovation ability	number of sports research instruments and equipment(+)	PCS	R_7_
		number of sports research projects(+)	PCS	R_8_

*“+” in parentheses indicates a positive index and “-” indicates an inverse index (Zhao et al., 2021).*

### The Selection of Evaluation Index of Regional Development Resilience of Sports in China

#### Driver Indexes

The development of the sports system is subject to the combined effects of the external and internal environment. The external environment is the necessary conditions that sports development must rely on, including economic, social, natural, and elements of other aspects (Chen, 2013). At the same time, the internal driver is also the driver of the self-organized development of the sports system, which is the key point to the development of the sports system’s resilience (Shao, 2015). It mainly refers to elements like human resources, material resources, financial resources, and scientific technology in the development of sports resilience. Before the sport system experiences perturbations, strong drivers, which is regarded as the prevention ability of the sport system, can effectively prevent the impact and perturbations on the sport system caused by internal and external environmental changes. Therefore, this study evaluated the driver in two dimensions: external driver and internal driver, and selects 7 indexes: gross national product, year-end population, disposable income per capita (R&D) personnel full-time equivalent, financial expenditure on culture, sports and media, number of practitioners in the sports industry and number of public sports facilities.

#### Pressure Indexes

The sports system, stimulated by internal and external environment drivers, would exert pressure directly or indirectly within the system, arousing the system’s internal structural element changes and buffering external impacts. This is regarded as an ability to buffer against impacts. This study will select four indexes in terms of economy, population society, and natural environmental pressure, which specify as GDP growth rate, natural population growth rate, unemployment rate, and green coverage rate of built-up areas (Ji et al., 2020).

#### State Indexes

Under the effect of “pressure,” the sports system deforms in various ways and presents different “states.” This state is the realistic performance of the resilience of sports development under drivers and pressures. It is also a kind of goal to be achieved by sports. This state is reflected in aspects of competitive sports, mass sports, and the sports industry. Therefore, this study selects four indexes: the number of athletes in excellent sports teams, the rate of national reaching standard for physical quality measuring, the number of sports social organizations, and sports lottery sales.

#### Impact Indexes

In the development of sports resilience, the internal and external environment will have impacts on the sport system, and in turn, changes in the sports system’s resilience state will also have various “impacts” on the surrounding ecological, economic, and social environment. In this study, four indexes are selected in terms of economy, society, and natural environments: the value-added of tertiary industry, the number of people employed in culture, sports, and entertainment industries, the death rate, and the forest coverage rate.

#### Response Indexes

Response refers to the effective measures and countermeasures taken by urban competitive sports subjects in the face of changes in the state of the urban competitive sports system. The process of sports system response is the process of system subjects deploying resources, responding to risk perturbations, and improving their own adaptivity and learning capabilities, which can generally be divided into three phases: early warning, restoration, and learning and innovation. The early warning ability of the sports system is mainly expressed as the ability to collect and transmit data, which is reflected in the level of network informationization of the sports system. Restorability refers to the ability of the system to recover from a crisis after a disturbance to the level before the system was disturbed (Bruneau et al., 2003). In other words, it is the subject’s ability to invest, integrate and mobilize the corresponding financial, material, and human resources. Learning and innovation ability refers to the system’s ability to not only restore to its original level after being disturbed but even to adapt to the new environment after the crisis, which is mainly reflected in the sports system’s level of scientific technology. Therefore, 8 indexes are selected in this study: the number of mobile Internet users, the number of graded athletes in development, the number of reserve sports talents, the number of youth sports clubs, the number of national physical quality monitoring stations, the number of public fit-trail projects, the number of sports research instruments and equipment, and the number of sports research projects.

## Analysis of the Resilience of China’s Sports Regional Development

### Research Design

In accordance with the new development philosophy and requirements of resilience development, this study attempted to build an evaluative index system for the resilience of China’s sports development with China’s 31 provinces, municipalities directly under the central government and autonomous regions as main research objects under the guidance of The CPC Central Committee’s proposals for formulating the 14th Five-Year Plan (2021–2025) for National Economic and Social Development and the Long-Range Objectives Through the Year 2035. All data, which range from 2013 to 2017, are collected from the China City Statistical Yearbook, China Sports Statistical Yearbook, and statistical communique of provinces and municipalities on national economic and social development over the years. Some index values are re-adjusted and calculated based on the statistics of the yearbook. The resilience of China’s sports development in different regions will be evaluated from the perspective of stages of sports development with the help of the DPSIR model, namely driver, pressure, state, impact, and response model.

### Research Methodology and Model

#### Determination of Evaluation Index Weights

(1) Normalize the matrix.

In collecting statistics for the evaluation index regarding the resilience of China’s sports development, different dimensions are found in 27 indexes. Therefore, we normalize each index via min-max normalization to avoid biases. Out of 27 indexes, only two of them have negative impacts, i.e., unemployment rate and death rate. Indexes with positive impact mean that when the value of one indicator increases, so does the resilience of sports development, whereas the smaller value of an index with negative impact means weaker resilience. The mathematical formulation for indexes with positive impact is


(1) $$x_{i\,j}^{\prime }=\frac{x_{i\,j}-\min x_{i\,j}}{\max x_{i\,j}-\min x_{i\,j}}$$

The mathematical formulation for indexes with negative impact is


(2) $$x_{i\,j}^{\prime }=\frac{\max x_{i\,j}-x_{i\,j}}{\max x_{i\,j}-\min x_{i\,j}}$$

$x_{i\,j}^{\prime }$ stands for the normalized value of indicator j for a region in the i year, *x*_*ij*_is the default value of indicator j for a region in the i year, *min*⁡*x*_*ij*_means the minimum value of index j while *max*⁡*x*_*ij*_the maximum value. i = 1, 2, …, 31. j = 1, 2, …, 27.

(2) Consider the weights based on the coefficient of variation.

To avoid subjectivity, the method of coefficient of variation is adopted to calculate weights for 27 indexes. The following are formulations for calculating the weight of each index:


(3) $$V_{j}=\frac{S_{j}}{{\bar{x}}_{j}}$$

(4) $$W_{j}=\frac{V_{j}}{\sum_{j=1}^{27}V_{j}}$$

${\bar{x}}_{j}$ is the mean value of indicator j, *S*_*j*_is the sample standard deviation of indicator j, *V*_*j*_is the coefficient of variation of indicator j, and is the weight of indicator j.

We add up the values of 27 indicators according to the weight of each indicator, meaning that a normalized weighted matrix is created via *W* = (0.047,0.030,0.020,0.062,⋯⋯,0.067,0.040,0.096,0.064) with *m* = 31, *n* = 27.


(5) $$R={\left(z_{i\,j}\right)}_{m\times n}={\left(W_{j}\,x_{i\,j}^{\prime }\right)}_{m\times n}$$

#### Evaluative Method Based on Technique for Order Preference by Similarity to an Ideal Solution Model

Technique for Order Preference by Similarity to an Ideal Solution (TOPSIS) model refers to a multi-criteria decision-making technique that makes the choice of the best alternative from among a finite set of decision alternatives in terms of multiple criteria (Zhao et al., 2017). TOPSIS provides an objective evaluation through the geometric distance (or similarity) between the chosen alternative and the ideal solution (both positive and negative) (Hwang and Yoon, 1981; Li et al., 2014).

Identify the positive and negative ideal solutions.

We identified the positive ideal solutions Z+ and negative ideal solutions Z-, where positive ideal solutions are the maximum of each column in R, whereas negative ideal solutions are the minimum.


(6) $$Z^{+}=\left(\max Z_{i\,1},\max Z_{i\,2},\cdots ,\max Z_{i\,n}\right)$$

(7) $$Z^{-}=\left(\min Z_{i\,1},\min Z_{i\,2},\cdots ,\min Z_{i\,n}\right)$$

Identify the distance from an alternative to the positive and negative ideal solutions, respectively.

We calculated the Euclidean distances ($D_{i}^{+}$ and $D_{i}^{-}$) from the positive ideal solution (Z+) and the negative ideal solution (Z-) of each alternative (every year of a region) respectively. The formulations are as follows:


(8) $$D_{i}^{+}=\sqrt{\sum_{i=1}^{m}{\left(\max Z_{i\,j}-Z_{i\,j}\right)}^{2}}$$

(9) $$D_{i}^{-}=\sqrt{\sum_{i=1}^{m}{\left(\min Z_{i\,j}-Z_{i\,j}\right)}^{2}}$$

(10) $$C_{i}=\frac{D_{i}^{-}}{D_{i}^{-}+D_{i}^{+}}$$

Calculate the comprehensive evaluation index

We calculated the relative closeness *C*_*i*_ (i.e., resilience) for each alternative with respect to positive ideal solutions according to formulation (10). The best alternative is one that is closer to a positive ideal solution and has a higher value *C*_*i*_, meaning greater resilience of sports development for that specific region. Later, we measure and rank the resilience of sports development in each province in China according to the statistics obtained.

### Result Analysis and Discussion

#### Analysis of the Integral Evaluation Results of the Resilience of Sports Regional Development in China

We calculated the resilience of sports development in five consecutive years (2013–2017) in 31 provinces, municipalities, and autonomous regions, as can be seen in Table 2. Overall, the result signified an upward trajectory in terms of resilience of sports development while certain provinces witnessed pronounced fluctuations. Jiangsu, Guangdong, and Zhejiang provinces have topped the lists from 2013 to 2017, ranking among the top three every year. Shandong provinces, despite slight fluctuations in ranking, boasted a steady increase. Moreover, major fluctuations happened in Hubei province in 2015 and 2017 with the index down by 26.25 and 31.88%, respectively. The result can be attributed to the decline in fixed-asset investment in culture, sports, and entertainment industries, in the natural growth rate of population, in the number of national fitness centers, youth sports clubs, fitness surveillance centers, and research on sports. Shanxi province experienced declining resilience of sports development because of less robust economic growth. Even though Shaanxi province, a landlocked province located in northwest China, is the gateway connecting the northwest, southwest, north, and central China and borders on eight neighboring provinces, municipalities, and autonomous regions, it receives less support from the reform and opening-up policy which has been led by the economic development of eastern, coastal areas. However, the ranking of Shaanxi province grew rapidly because growing western and central regions and regional development strategies have led to thriving economic development in Shaanxi province, thus increasing the fixed-asset investment in culture, sports and entertainment industries, the investment in sports personnel and materials, and the support for sports technology.

**TABLE 2:** Scores and rankings of the resilience of sports development in each region in China.

Region/Year	2013	2014	2015	2016	2017
Jiangsu	0.4004	0.4295	0.3933	0.4235	0.4427
	1	1	2	3	2
Guangdong	0.3563	0.3995	0.3937	0.4200	0.4795
	2	2	1	4	1
Zhejiang	0.3562	0.3296	0.3775	0.3855	0.4012
	3	4	3	5	3
Shandong	0.3234	0.3455	0.3468	0.3795	0.3796
	4	3	4	6	4
Hubei	0.2003	0.3212	0.2369	0.4423	0.3013
	10	6	7	2	5
Henan	0.2250	0.3294	0.2429	0.2621	0.2803
	8	5	6	8	6
Beijing	0.2488	0.2242	0.3059	0.2291	0.2645
	6	9	5	10	9
Hebei	0.2710	0.2306	0.2218	0.2675	0.2769
	5	7	9	7	7
Shanxi	0.1798	0.1601	0.1612	0.4458	0.2763
	14	17	18	1	8
Sichuan	0.2113	0.2139	0.2237	0.2284	0.2292
	9	11	8	11	12
Hunan	0.1799	0.1899	0.2097	0.2392	0.2637
	13	14	10	9	10
Fujian	0.1845	0.2022	0.2083	0.2178	0.2337
	12	12	11	12	11
Liaoning	0.1932	0.2249	0.1955	0.1924	0.1311
	11	8	12	16	20
Anhui	0.1609	0.1941	0.1955	0.2133	0.2082
	16	13	13	13	14
Jiangxi	0.2305	0.1723	0.1773	0.1688	0.2009
	7	15	15	18	16
Shanghai	0.1786	0.1701	0.1729	0.2005	0.2142
	15	16	17	15	13
Guangxi	0.1597	0.2217	0.1775	0.1698	0.1741
	17	10	14	17	17
Yunnan	0.1490	0.1511	0.1546	0.2031	0.1740
	18	18	19	14	18
Heilongjiang	0.1321	0.1491	0.1771	0.1638	0.1656
	23	19	16	19	19
Neimeng	0.1075	0.1354	0.1331	0.1661	0.2060
	29	20	20	20	15

*Source: Author’s calculations. Limited by space, only the top 20 provinces and autonomous regions are listed here. The bottom 11 provinces and autonomous regions, including Tianjin, Shanxi, Jilin, Hainan, Chongqing, Guizhou, Tibet, Gansu, Qinghai, Ningxia, and Xinjiang, are not included in the list.*

**FIGURE 1:**
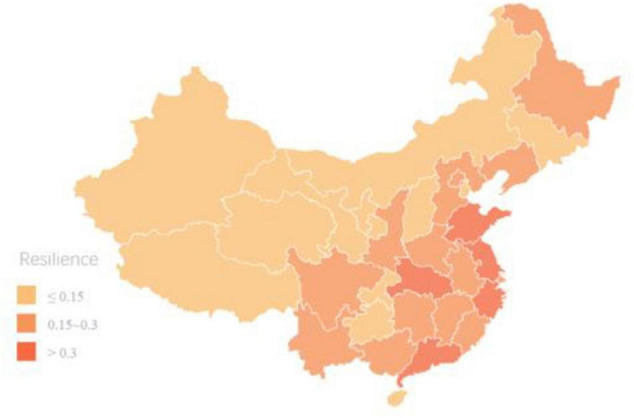
Cluster distribution of average resilience level of sports regional development in China.

Table 3 presents the result of cluster analysis after the average of comprehensive evaluation indices of each region between 2013 and 2017 is calculated. As can be seen in Figure 1, compared with those in the west, regions in east China enjoyed higher resilience of sports development, serving as a driver in China’s sports development.

**TABLE 3:** Clustering of average scores based on the integral development resilience assessment.

Scores	The first level (Average Score ≥ 0.3)	The second level (0.15 ≤ Average Score < 0.3)	The third level (0 ≤ Average Score < 0.15)
Region	Jiaangsu, Guaangdong, Zhejiang, Shansong, Hubei	Henan, Beijing, Hebei, Shanxi, Sichuan, Hunan, Fujian, Liaoning, Anhui, Jiangxi, Shanghai, Guangxi, Yunnan, Heilongjiang	Neimeng, Shanxi, Chongqing, Xinjiang, Guizhou, Hainan, Jilin, Tianjin, Gansu, Tibet, Ningxia, Qinghai
Location	Almost all in the eastern region	Most are in the eastern and central regions	Most of them are in the western region

#### Evaluation and Analysis of Each Subsystem of the Level of Resilient Development of Sports in Each Region in China

(1) Driving force subsystem

**FIGURE 2:**
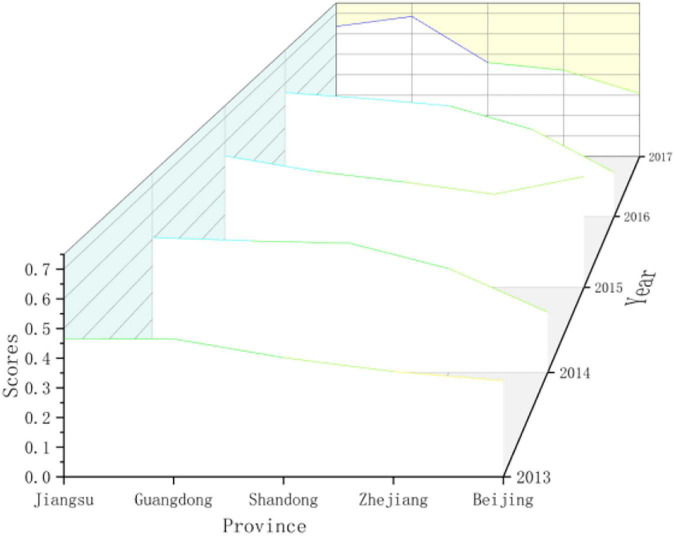
Comparing the scores of the sports development resilience driving force subsystem in some regionals in China.

Firstly, we selected the top provinces in the driving force score for comparison and analysis, as shown in Figure 2. On the whole, Jiangsu and Guangdong’s driving force scores were higher than those of Shandong, Zhejiang, and Beijing, and they were showing a clear upward trend. Although Shandong’s driving force scores in 2015 and 2017 declined compared with the previous year, it is still relatively high compared to other provinces. Zhejiang’s driving force score has risen steadily and is relatively stable. However, Beijing’s driving force score shows a “W” trend, with large fluctuations. In 2014 and 2016, the driving force score of Beijing dropped to 0.2268 and 0.1993, a decrease of 29.95 and 56.46%, respectively, from the previous year. The development trend of sports is unstable.

**FIGURE 3:**
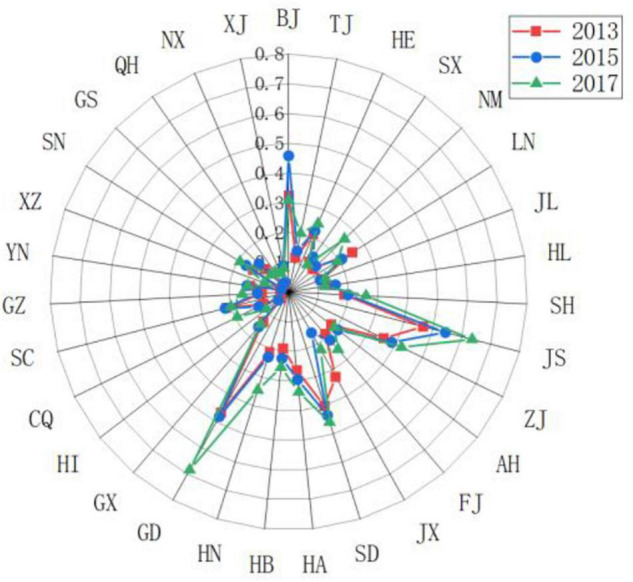
Comparing the scores of the sports development resilience driving force subsystem in various regionals in China.

Secondly, we used the years 2013, 2015, and 2017 to compare and analyze the changing trends of the driving force levels of various provinces in China, as shown in Figure 3 (Zhu et al., 2020). It was found that Guangdong, Jiangsu, and Inner Mongolia have changed a lot in driving force. Guangdong and Jiangsu had steadily increased their driving force scores. Due to their own geographical location and environmental advantages, as well as the support of national policies, they had shown huge development space and potential. In recent years, under the influence of policies such as the Great Western Development, Inner Mongolia has gradually strengthened its driving force and has certain development potential.

In summary, the overall driving force scores of most provinces in China are on the rise, indicating that the pressure on the sports system caused by China’s social and economic development is gradually decreasing, and a good driving effect has been achieved.

**FIGURE 4:**
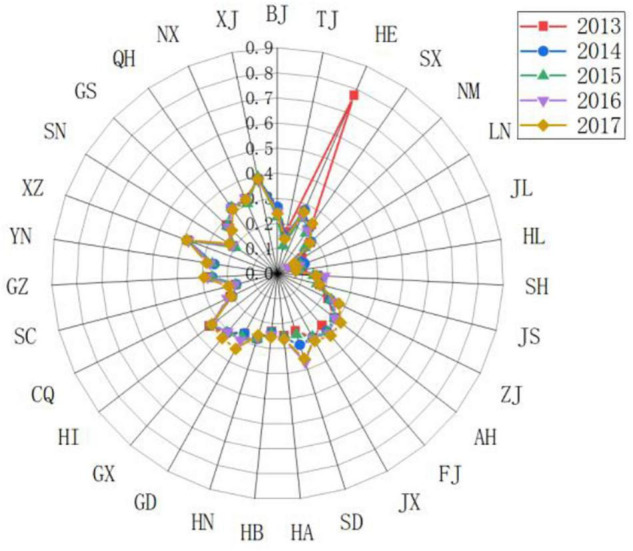
Comparing the scores of the sports development resilience pressure subsystem in various provinces in China.

(2) Pressure subsystem:

As shown in Figure 4, during the period of 2013–2017, the scores of the pressure subsystem of sports resilience development in China’s provinces did not change much. Among them, Zhejiang, Anhui, Fujian, Hubei, Guangdong, and Sichuan’s pressure scores increased slightly. Their pressure subsystems are at a relatively good level by stimulating GDP growth, increasing the natural population growth rate, improving social stability, and improving the natural environment. Liaoning, Jilin, and Heilongjiang are faced with slow GDP growth, population decline, over-exploitation of natural resources, and environmental damage, so their pressure subsystem scores are low and have a slight downward trend. Other provinces like Beijing, Hebei, Hainan, Shanxi, Henan, Guangxi, Gansu, Qinghai, Ningxia, and Xinjiang are affected by the western development policy, which has driven their GDP to grow rapidly. At the same time, they have the advantage of population growth, which puts them under pressure. The system is at a good level, but the development is unstable, showing small fluctuations.

(3) Status subsystem

**FIGURE 5:**
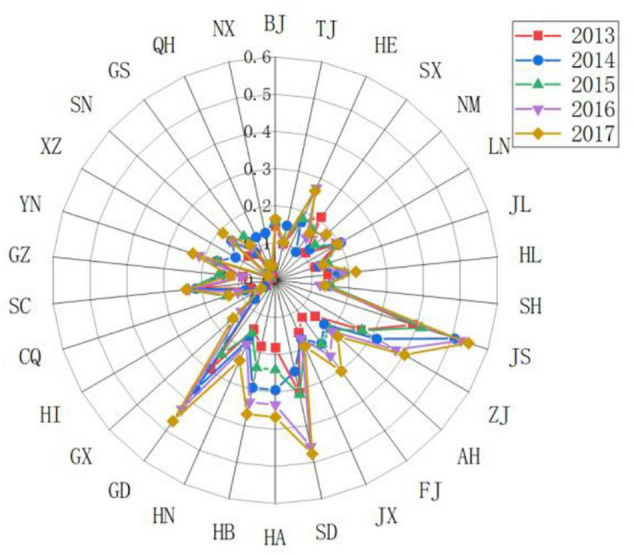
Comparing the scores of the sports development resilience status subsystem in various provinces in China.

Under the dual effects of driving force and pressure, as shown in Figure 5, Jiangsu, Shandong, Guangdong, and other eastern regions are actively cultivating emerging sports industries, formulating sports development strategies and systems, and implementing regional sports plans under the rapid economic development. Which effectively eliminates part of the negative impact and maintains a better state of sports development. When Beijing, Tianjin, Inner Mongolia, Jilin, Shanghai, Hainan, Guizhou, Tibet, Guizhou, Qinghai, Ningxia, and Xinjiang face the problems caused by the development of sports, the effectiveness of governance measures taken is not obvious, resulting in insufficient effective supply of sports resources. Insufficient talents and other problems appear, the existing governance measures cannot effectively improve the state of sports development, so the state subsystem scores are at a low level.

Generally speaking, the current state of sports development in the eastern and central provinces of China is relatively good. Although the state of sports development in the western region is on the rise, sports resources and environmental issues have not been effectively resolved. In order to improve the state of sports development, sports resources, and sports environment, it needs to be further rationally utilized, managed and protected, and further improved.

(4) Influence subsystem

**FIGURE 6:**
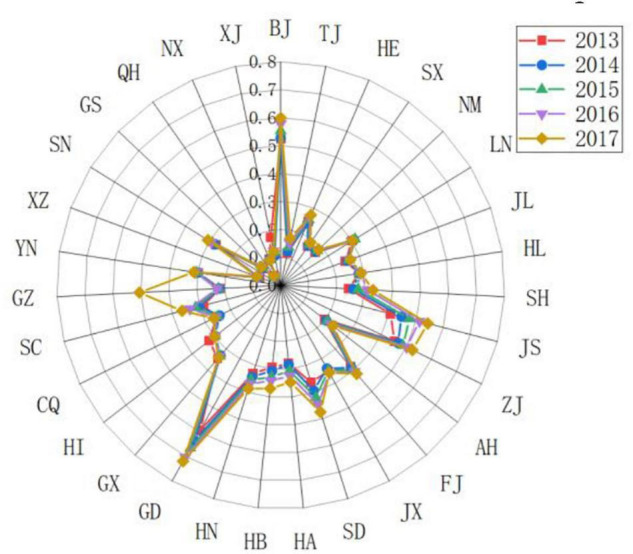
Comparing the scores of the development of the sport resilience influence subsystem in various provinces in China.

Judging from the impact of the economy, society, population, and natural resources of each province, the impact subsystem of Guangdong, as shown in Figure 6, Beijing, and Jiangsu is at a relatively high level. Sports promote the value of the tertiary industry and increase the employment population. Health promotion and improvement of environmental resource utilization have significant effects. Tianjin, Shanxi, Inner Mongolia, Anhui, Tibet, Gansu, Qinghai, Ningxia, and Xinjiang have been greatly negatively affected, making the impact subsystem at a low level. Under the background of globalization, the sports industry has not been fully promoted. Development has failed to give full play to the role of sports social organizations in national fitness and cultivating competitive sports talents, resulting in a low level of influencing subsystems. Therefore, it is necessary to fully improve the sports industry policy, optimize the development environment, release development potential, and then stimulate economic growth and promote employment. At the same time, implement national fitness activities, strengthen the construction of green sports projects such as sports parks, improve the local natural resource environment, and further promote society’s various developments.

(5) Response subsystem

**FIGURE 7:**
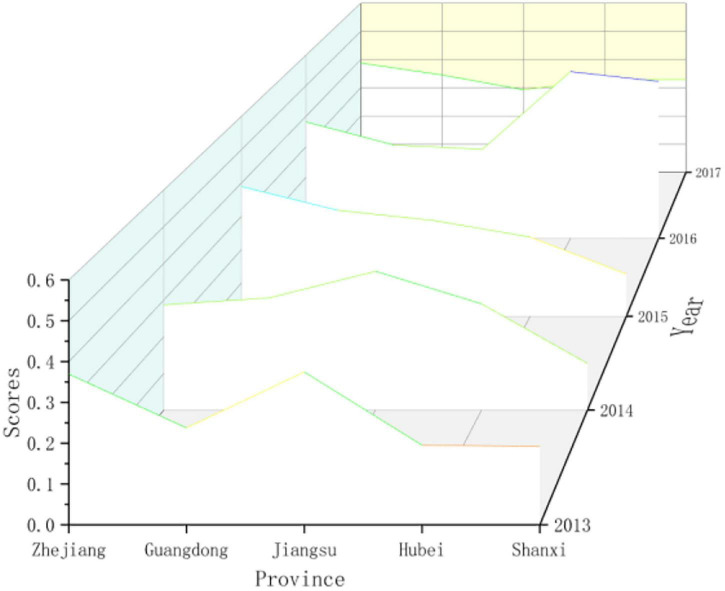
Comparing the scores of the sports development resilience response subsystem in some provinces in China.

**FIGURE 8:**
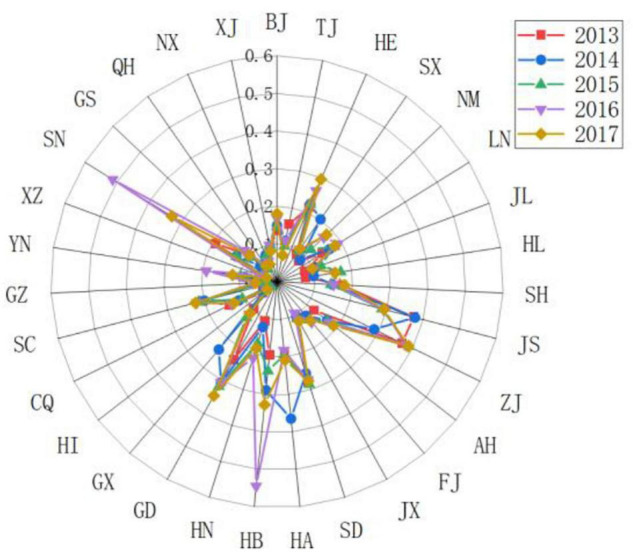
Comparing the scores of the sports development resilience response subsystem in various provinces in China.

Different provinces have different response levels to the driving force and pressure of sports development. As shown in Figures 7, 8, Zhejiang, Guangdong, Jiangsu, Hubei, and Shaanxi have achieved better governance results. In terms of sports talent training, vigorously develop the number of level athletes, strengthen the training of sports reserve talents, and vigorously establish youth sports clubs. In terms of national fitness, strengthen the construction of national fitness monitoring stations and national fitness path projects. In terms of technological innovation, we invested in sports technology equipment and strengthened sports project research to improve the level of sports technology innovation and promoted higher scores in response subsystems. The scores of response subsystems in Zhejiang, Guangdong, and Jiangsu remained within the range of 0.3–0.6. Various sports management Policies and measures have achieved good results. The sports management policies and measures implemented in Beijing, Tianjin, and Shanghai have achieved low response effects. Sports governance capabilities and sports technology innovation levels are difficult to adapt to the rapid growth of the sports industry and the demand for national fitness in Beijing, Tianjin, and Shanghai in recent years. Investment in governance and sports technology innovation needs to be increased. Guizhou, Yunnan, Tibet, Gansu, Qinghai, Ningxia, and western Xinjiang have relatively low scores in response subsystems, and there are still relatively large areas of informatization, investment in sports resources, training of sports talents, construction of national fitness projects, and innovation in sports science and technology. The problem is that although some provinces have adopted some measures and achieved some results, the overall situation is still not ideal and still faces severe tests.

## Obstacle Factors Affecting the Resilience of Sports Regional Development in China

### Research Design

Based on the evaluation of the development level of sports resilience in China from 2013 to 2015. We introduce the obstacle degree model to diagnose and identify the main obstacle factors that affect the resilience of sports development in China during 2013–2015. Then the first five major factors will be analyzed.

### Research Method

The obstacle model can provide some suggestions for the formulation and adjustment of sports development policy by analyzing and diagnosing the obstacle factors that affect the resilience level of sports development in each province (Pan et al., 2019). The factor contribution degree is represented by the weight wj of a single index. Index deviation degree Ij refers to the difference between a single index and the target expressed as the difference between the standardized value of each indicator and 1, and $x_{j}^{\prime }$ is standardized data. Obstacle degree Oj means the degree to which each indicator or criterion layer factors affect the development of regional sports resilience in China (Huang et al., 2020). The obstacle degree is calculated as follows:


(11) $$I_{j}=1-x_{j}^{\prime }$$

(12) $$O_{j}=\frac{I_{j}\times w_{j}}{\sum_{j=1}^{n}I_{j}\times w_{j}}\times 100\%$$

According to the calculation formula of the obstacle degree, the influence degree of each index on the development toughness of China’s sports regions is obtained.

### Result Analysis and Discussion

#### The Main Obstacle Factors Affecting the Resilience of Sports Regional Development in China

It can be seen from Table 4 that the obstacles (frequency greater than 5) affecting the development of sports resilience in China from 2013 to 2015 are in order as follows: sports scientific research equipment (R7), the number of national fitness monitoring stations (R5), the number of national fitness activity centers (D7), the full-time equivalent of (R&D) personnel (D4), and the number of sports scientific research projects (R8).

**TABLE 4:** The main obstacle factors and obstacle degree in the evaluation index layer of sports regional development resilience in China from 2013 to 2015.

Year	Type	Order of indexes				
		1	2	3	4	5
2013	obstacle factors	**R_7_**	**R_5_**	**P_1_**	**D_7_**	**D_4_**
	obstacle degree	10.03%	8.34%	7.01%	6.84%	6.63%
2014	obstacle factors	**S_1_**	**R_7_**	**R_8_**	**D_4_**	**R_5_**
	obstacle degree	10.00%	8.92%	8.81%	6.71%	6.08%
2015	obstacle factors	**R_7_**	**R_5_**	**D_1_**	**D_7_**	**D_4_**
	obstacle degree	9.29%	8.36%	7.99%	7.73%	7.22%
2016	obstacle factors	**R_7_**	**R_5_**	**D_7_**	**R_8_**	**D_4_**
	obstacle degree	12.24%	8.54%	7.59%	7.27%	6.95%
2017	obstacle factors	**R_7_**	**R_5_**	**D_7_**	**R_8_**	**D_4_**
	obstacle degree	10.10%	9.61%	8.80%	7.47%	7.22%

In terms of time periods, in 2013 the top five factors affecting the resilience of China’s sports development were sports scientific research equipment (R7) the number of national fitness monitoring stations (R5), GDP growth (P1), the number of national fitness activity centers (D7), and the full-time equivalent of (R&D) personnel (D4). It shows that the development level of sports science and technology in China is weaker at this stage, and the national investment in sports science and technology is lower, the national construction project is not well carried out, and the investment in national fitness testing center and fitness activity center is not enough. In addition, the decline in GDP growth rate has also increased the pressure on the sports system and has also hindered the development of sports to some extent.

In 2014, the top five factors affecting the resilience of China’s sports development were the number of athletes in elite sports teams (S1), sports scientific research equipment (R7), the number of sports science research projects R8, full-time equivalent (R&D) personnel (D4), and the number of national fitness monitoring stations (R5). Elite sport is a demonstration of the strength of a country and a nation. After experiencing the glory of the 2018 Beijing Olympic Games, elite sports in China were declining to some extent. Elite athletes are the most critical human resources for the development of national elite sports. At this stage, there were problems in the cultivation of elite athletes, which became the most important obstacle to the resilience of China’s sports development. In addition, the national investment in sports science and technology is still low, showing a bold form of development. National physique monitoring, meanwhile, has not received enough attention. All these have influenced the development of sports.

In 2015, sports scientific research equipment (R7) and the number of national physical fitness monitoring stations (R5) are still the first and second obstacles affecting the resilience of sports development in China. It shows that the level of sports science and technology still needs to be improved. In addition, GDP (D1) became the third obstacles factor. It shows that national economic development is still the main factor that restricts the development of sports. Thirdly, the number of national fitness centers (D7) and the full-time equivalent (R&D) personnel (D4) are still the main obstacles affecting the development of sports in China. This also shows that the country’s investment in national fitness and science and technology still needs to be improved.

In 2016 and 2017, the main obstacles affecting the resilience of sports development in China were sports scientific research equipment (R7), the number of national fitness monitoring stations (R5), the number of national fitness activity centers (D7), the number of sports science research projects, the full-time equivalent of R8 (R&D) personnel, and (D4). This also shows that technology is still the primary productive force in the development of sports, especially in the new era. Under the new development concept, science and technology, especially digital sports, will inject new impetus into the development of national health and sports. At present, although China has made great progress in sports science and technology, the basic research ability of sports science and technology is insufficient, the number of high-level sports science and technology laboratories is short, and the quality of research results and the ability of transformation need to be improved. Although, sports has achieved rapid development through the promulgations of national strategies such as “Sports power,” and “Healthy China,” it still cannot meet the growing material and cultural needs of the people. It was still a lack of Sports infrastructure. Therefore, China should continue to promote the national fitness project construction, especially increase the investment in national fitness infrastructure, to promote the vigorous development of sports in China.

#### The Main Obstacle Factors Affecting Each Subsystem of the Resilience of Sports Regional Development in China

By calculating the obstacle degree of the five subsystems, the results are shown in Table 5. The response subsystem is the main classification index obstacle factor that affects the resilience improvement of sports development in China, followed by the driving force subsystem and the state subsystem. The order is: response > driving force > state > impact > pressure. Therefore, in order to further improve the resilience of sports development in China, in the context of the changing complex environment and the increasing pressure of sports development environment. We should focus on the response subsystem, balancing the driving force subsystem and the state subsystem. Establishing the development concept with national fitness as the core, making full use of science and technology innovation, coordinating the development of national fitness and elite sports, and developing the sports industry. In this way, we can alleviate the negative effects of sports development.

**TABLE 5:** The Subsystem obstacle degree of sports regional development resilience in China from 2013 to 2015.

Year	Obstacle degree				
	Driving force subsystem	Pressure subsystem	State subsystem	Impact subsystem	Response subsystem
2013	27.52%	9.73%	11.10%	9.93%	41.71%
2014	27.19%	3.71%	17.37%	10.26%	41.47%
2015	33.66%	4.54%	9.60%	10.60%	41.60%
2016	29.34%	4.54%	10.82%	10.23%	45.07%
2017	31.07%	4.21%	10.52%	10.73%	43.48%

## Analysis of Spatial Correlation of Sports Development in China’s Different Regions

### Research Design

According to the First Law of Geography, everything is related to everything else, but near things are more related than distant things (Pan et al., 2020). This study will analyze the spatial correlation of the resilience of sports development in 31 provinces, municipalities, and autonomous regions in China from 2013 to 2017 to figure out whether a spatial correlation exists. Spatial autocorrelation can be classified into global spatial autocorrelation and local spatial autocorrelation. Measures of global spatial autocorrelation include Moran’s I, General G; whereas methods of local spatial autocorrelation mainly contain LISA, local G and the Moran scatter plot. Global Moran’s I and the Moran scatter plot will be adopted to study the spatial correlation of the resilience of sports development among different regions in China.

### Research Method

In this paper, the Global Moran’s I and local Moran’s I t are used to study the spatial correlation of the development resilience levels of sports regions in China. Global Moran’s I, the most common indicator of spatial autocorrelation, is used to analyze the spatial homogeneity of an overall region and measure how one feature of different variables is similar or correlated to others surrounding it (Wang and Ge, 2012). Moran’s I is defined as


(13) $$I=\frac{\sum_{i=1}^{n}\sum_{j=1}^{n}w_{i\,j}\,\left(X_{i}-\bar{X}\right)\,\left(X_{j}-\bar{X}\right)}{s^{2}\,\sum_{i=1}^{n}\sum_{j=1}^{n}w_{i\,j}}$$

In particular, _*x_ij_*_ stands for the value of resilience of sports development in each region; _*w_ij_*_ is the normalized spatial weights matrix.

Since global Moran’s I cannot visualize the similarity or difference in terms of spatial gathering in a certain locality, we calculated the local Moran’s I for 31 provinces, municipalities, and autonomous regions between 2013 and 2017 and then analyzed the local spatial correlation with Moran scatter plot (Yu et al., 2016). The formulation of local Moran’s I is presented as follows:


(14) $$I_{i}=\frac{\left(x_{i}-\bar{x}\right)}{S^{2}}\,\sum_{j=1}^{n}w_{i\,j}\,{\left(x_{j}-\bar{x}\right)}^{2}$$

### Result Analysis and Discussion

#### Analysis of Global Spatial Correlation

Table 6 presents the result of global Moran’s I about the resilience of China’s sports development. The value of Moran’s I was all above 0 from 2013 to 2017. Except for 2016, the global Moran’s I value of each year passed the test of significance (10% threshold). As for the *z*-score, 2013 witnessed a significant positive correlation between location and the resilience of sports development with the *z*-score exceeding 1.95. The same result can be seen in the year 2014 with a z-score over 1.65. The *z*-score from 2015 to 2017 indicated that the spatial autocorrelation is insignificant with no obvious clustering. In addition, the value of Moran’s I from 2013 to 2017 was 0.251, 0.185, 0.147, 0.052, and 0.137, respectively. The unstable and downward trend indicated that the spatial autocorrelation was declining.

**TABLE 6:** Global Moran’s I about the Resilience of China’s Sport Region Development from 2013 to 2017.

Year	Moran’s I	z-score	p-value
2013	0.251	2.429	0.008
2014	0.185	1.854	0.032
2015	0.147	1.535	0.062
2016	0.052	0.718	0.236
2017	0.137	1.453	0.073

#### Analysis of Local Spatial Correlation

**FIGURE 9:**
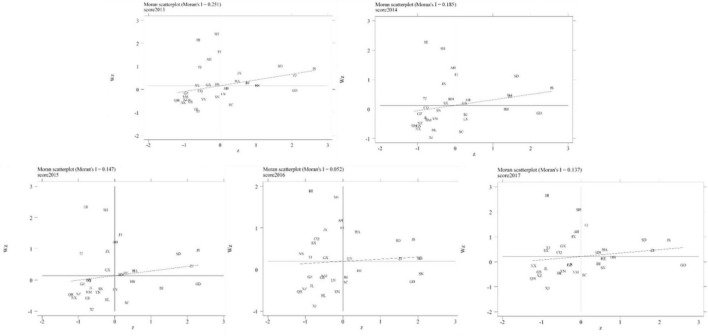
Moran Scatter Plot of the resilience of China’s Sports Region Development from 2013 to 2015.

The Moran scatter plot is decomposed into four quadrants, corresponding with high-high (H-H, the upper-right quadrant), low-high (L-H, the upper-left quadrant), high-low (H-L, the lower-right quadrant), and low-low (L-L, the lower-left quadrant) spatial correlation. Figure 9 visualizes the local spatial correlation.

(1) H-H quadrant: A province in the H-H quadrant means it enjoys higher resilience of sports development, so do its surroundings and neighboring provinces, showing a small spatial difference. Jiangsu, Zhejiang, Shandong, Jiangxi, and Henan provinces, as well as Beijing Municipality, belonged to the H-H quadrant in 2013, among which Jiangsu, Zhejiang, Shandong, and Henan provinces have always been in this quadrant. The result indicated that cluster development on a large scale has been established among the abovementioned provinces and their neighborhood, mainly in the eastern region with strong economic growth, advanced IT application, abundant sports resources, large public investment in sports, and full-fledged sports infrastructure. In terms of time span, Hebei, Guangxi, Fujian, and Hunan provinces have been uplifted from H-L to H-H quadrant with each passing day, reflecting a stronger spatial correlation with neighboring provinces. For example, Hebei province’s development was made possible by the impact from the neighboring provinces of Shandong and Henan which enjoy rapid development in sports, while Fujian benefited from the neighboring provinces of Zhejiang. All these led to a coordinative development with the neighboring provinces and at the same time sustained self-development (Xu et al., 2021).
(2) L-H quadrant: A province in the L-H quadrant means it has lower resilience of sports development whereas its surroundings and neighboring provinces boast higher resilience. Tianjin, Shanghai municipalities, and Hainan, and Anhui provinces belonged to the L-H quadrant in 2013, but Jiangxi province was downgraded from H-H to L-H quadrant from 2014 to 2017, showing the influence of surroundings was weak despite strong development in Jiangxi. On the contrary, Fujian province was upgraded from L-H to H-H quadrant gradually, showing that Fujian province has strengthened its ties with neighboring provinces while improving its own resilience of sports development, thus forming a robust cluster development with its neighbors.
(3) H-L quadrant: A province in the H-L quadrant means it boasts higher resilience of sports development whereas its surroundings and neighboring provinces have lower resilience. Guangdong, Sichuan, Liaoning, and Hubei provinces belonged to the H-L quadrant in 2013, meaning that focus on self-development in sports brought greater resilience of development. However, due to the slow growth of neighbors, little cluster development can be found in the provinces mentioned above. Starting from 2014, Beijing municipality has downgraded from H-H to H-L quadrant, demonstrating a declining correlation with its surroundings due to weakening radiation impact. In contrast, the upgrading of Shaanxi province from L-H to H-L quadrant showed that with sustained economic development, this province has paid high attention to sports development, improving the resilience while the development of the slow sport caused restricted resilience.
(4) L-L quadrant: A province in the H-H quadrant means it enjoys higher resilience of sports development, as do its surroundings and neighboring provinces. In 2013, the L-L quadrant was home to 12 provinces and autonomous regions including Qinghai, Ningxia, Gansu, Guizhou, and Xinjiang. The same result can be seen even in 2017. Most of them, located in the western region, grew slowly in terms of the economy because of the location, natural environment, and other conditions. In addition, the long-distance between provinces in the west and those in the east resulted in weak radiation and made it difficult to obtain greater resilience and form cluster development on a large scale, leading to slow sports development.

## Research Results and Suggestions

### Research Results

(1) Obtain the weight of the evaluation index. The study, in the spirit of new development philosophy, analyzed features of China’s sports development, created an evaluative index system measuring the resilience of China’s sports development in different regions from five dimensions of “driver, pressure, state, impact and response” (DPSIR model) according to the dynamism of China’s sports development and determined the evaluation index weights based on the coefficient of variation.
(2) Measured the resilience level of sports development in each region. With the help of the TOPSIS model (Zhu et al., 2016), we calculated the resilience of sports development from 2013 to 2017 in 31 provinces, municipalities, and autonomous regions, finding out an upward trajectory in terms of resilience of sports development overall while certain provinces witnessed pronounced fluctuations. Compared with localities in the west, regions in the east enjoy higher resilience of sports development. Among different sub-systems, the score of driver and impact showed an upward trend while that of pressure remained basically unchanged with slight fluctuations. Also, the score of impact bounced back rapidly after a temporary slowdown while that of response declined after a gradual increase.
(3) Rank the obstacles degree. Obstacles affecting the resilience of China’s sports development identified by the Obstacle Degree Model (ODM) include (in the order of scores): equipment for sports science, fitness surveillance center, national fitness center, the full-time equivalent (FTE) of R&D personnel, and amounts of research on sports. The order of obstacle degree is response > driver > state > impact > pressure.
(4) Analyzed the spatial correlation of the resilience of sports development. Analysis of spatial correlation of the resilience of sports development in 31 provinces, municipalities, and autonomous regions in China from 2013 to 2017 proved a strong cluster in terms of resilience in 2013 and 2014 while no obvious cluster was found from 2015 to 2017. Moreover, spatial autocorrelation of the resilience of China’s sports development continues to weaken. With regard to the local Moran scatter plot, great differences remain in different regions with the H-H quadrant home to economically developed provinces in the east and the L-L quadrant containing underdeveloped provinces, municipalities, and autonomous regions in the west with low resilience.

### Suggestions

The Fifth Plenary Session of the 19*^th^* CPC Central Committee adopted *The CPC Central Committee’s proposals for formulating the 14th Five-Year Plan (2021–2025) for National Economic and Social Development and the Long-Range Objectives Through the Year 2035*, stating that “We must ensure the new development philosophy is applied in every stage and aspect of development.” To improve the resilience of China’s sports development in an all-around way, we must take the new development philosophy as the guiding principle. First, we should stick to innovation-driven development to fully upgrade the resilience of China’s sports development. Since innovation is a powerful engine propelling sports development in the new era, it is necessary to boost innovation in different dimensions of sports development, in particular the innovation of sports technology. Moreover, effort should be made to increase input in human capital and funding for innovative development while following through the strategy of innovation-driven development so as to improve the efficiency of innovative sports development in an all-around way.

Second, we should adhere to the principle of coordinated development to promote the overall and balanced development of sports. Efforts should be made to expand national fitness facilities in a bid to greatly promote national fitness and health while enhancing coordinated development of competitive sports and fitness, thus improving people’s health while looking for and cultivating talents for competitive sports. At the same time, we should strengthen information exchanges and cooperation among different localities by establishing a highly efficient coordinative mechanism and a sound and stable cooperation platform to promote the free flow of sports resources in various regions. Furthermore, all regions should share resources, draw on each other’s strengths, and deepen coordination and cooperation so as to achieve win-win results. It is imperative to strengthen measures to reach a new stage in the large-scale development of the western region; help the central region rise by tapping into local strengths; support the eastern region in taking the lead in pursuing optimal development through innovation. To this end, we need to put in place new, effective mechanisms to ensure coordinated development of different regions.

Lastly, we should promote shared development so as to deliver benefits for all in an equal way. All provinces, municipalities, and autonomous regions should carry out institutional reforms of sports development, explore new policy measures and cooperation methods, and focus on closing the gap among different regions in terms of sports development. Regions with higher resilience of sports development should not only ensure sustained growth but also exert a greater positive impact on surrounding areas, fully improving the resilience of sports development in other regions. In addition, regions with inadequate resilience of sports development should seize new opportunities brought by the policies such as China Western Development and integrated development of sports among regions, learn from the experience of developed regions so as to achieve greater development.

## Data Availability Statement

The original contributions presented in the study are included in the article/Supplementary Material, further inquiries can be directed to the corresponding author/s.

## Author Contributions

JZ designed the research and wrote the manuscript. J-RG and J-BL undertook the statistical analysis and graphical representation of the data. YW and SZ aided in reference collection and summary as well as participated in the manuscript preparation. BS directed the research process and revised the draft. All authors who designed this study contributed to the article and approved the final manuscript.

## Conflict of Interest

The authors declare that the research was conducted in the absence of any commercial or financial relationships that could be construed as a potential conflict of interest.

## Publisher’s Note

All claims expressed in this article are solely those of the authors and do not necessarily represent those of their affiliated organizations, or those of the publisher, the editors and the reviewers. Any product that may be evaluated in this article, or claim that may be made by its manufacturer, is not guaranteed or endorsed by the publisher.
